# 

**Published:** 1820-04

**Authors:** 


### MONTHLY CATALOGUE OF MEDICAL BOOKS.

Cases of Tic Douloureux, successfully treated. By Benjamin Hutchinson, Member of the Royal College of Surgeons of London, &c. 8vo. 3s. 6d.

A History of the Epidemic Fever which prevailed in Bristol during the Years 1817, 1818, and 1819, founded on Reports of St. Peter's Hospital and the Bristol Infirmary. By J. C. Pritchard, Esq. 8vo. 5s.

Medical Notes on Climates, Diseases, Hospitals, and Medical Schools, in France, Italy, Switzerland; comprising an Inquiry into the Effects of a Residence in the South of Europe in Cases of Pulmonary Consumption, &c. By James Clark, &c. 8vo. 7s.

A Succinct Account of the Typhous or Contagious Fever of this Country. By Thomas Bateman, M.D. F.L.S. 8vo. 6s. boards.

